# Structural and Functional Characterization of β−lytic Protease from *Lysobacter capsici* VKM B−2533^T^

**DOI:** 10.3390/ijms232416100

**Published:** 2022-12-17

**Authors:** Alexey Afoshin, Svetlana Tishchenko, Azat Gabdulkhakov, Irina Kudryakova, Inna Galemina, Dmitry Zelenov, Elena Leontyevskaya, Sofia Saharova, Natalya Leontyevskaya (Vasilyeva)

**Affiliations:** 1 Laboratory of Microbial Cell Surface Biochemistry, G.K. Skryabin Institute of Biochemistry and Physiology of Microorganisms, FRC PSCBR, Russian Academy of Sciences, 5 Prosp. Nauki, 142290 Pushchino, Russia; 2 Institute of Protein Research, Russian Academy of Sciences, 4 Institutskaya Str., 142290 Pushchino, Russia; 3 FSBEIHE Pushchino State Institute of Natural Sciences, 3 Institutskaya Str., 142290 Pushchino, Russia; 4 Faculty of Chemical Technology and Biotechnology, Moscow Polytechnic University, 38 Bolshaya Semyonovskaya Str., 107023 Moscow, Russia

**Keywords:** β−lytic protease, *Lysobacter capsici*, bacteriolytic enzymes, Blp crystal structure, LasA, MRSA, antimicrobial drugs, M23A protease, bacteriolytic activity

## Abstract

The crystal structure of the *Lysobacter capsici* VKM B−2533^T^ β-lytic protease (Blp), a medicinally promising antimicrobial enzyme, was first solved. Blp was established to possess a folding characteristic of the M23 protease family. The groove of the Blp active site, as compared with that of the LasA structural homologue from *Pseudomonas aeruginosa*, was found to have amino acid differences. Biochemical analysis revealed no differences in the optimal reaction conditions for manifesting Blp and LasA bacteriolytic activities. At the same time, Blp had a broader range of action against living and autoclaved target cells. The results suggest that the distinction in the geometry of the active site and the charge of amino acid residues that form the active site groove can be important for the hydrolysis of different peptidoglycan types in target cells.

## 1. Introduction

β−Lytic protease (Blp), together with α−lytic protease, was first described in 1965 [1], more than forty years after the discovery of the first bacteriolytic enzyme, lysozyme [2]. Lysozymes have been found in all living organisms and viruses, but α- and β−lytic proteases are currently known only in *Lysobacter* bacteria. The most lytically active but least studied among these three enzymes is Blp. The specificity of its action against living Gram−positive pathogens evokes much interest in this enzyme as an active basis for the creation of antimicrobial drugs.

Blp was first isolated from the bacterium *Myxobacter* 495 [1]. This bacterium is classified now as *L. enzymogenes*. Since then, this enzyme has been isolated from other *Lysobacter* bacteria: *L. enzymogenes* M497−1, *Lysobacter* sp. IB−9374, and *L. capsici* VKM B−2533^T^ [3,4,5]. The isolated enzymes have been characterized to various degrees [3,4,6]. In the peptidoglycan of target cells, the enzyme digests the bonds in the interpeptide bridge [4]. Recently, we have isolated a Blp from the culture fluid of *L. capsici* VKM B−2533^T^ and characterized it [5,6]. Apart from hydrolysing substrates for proteases, the enzyme is highly efficient in digesting the peptidoglycan of living *Micrococcus luteus* Ac−2230^T^ and *Staphylococcus aureus* 55 MRSA cells. Its use as a basis for the creation of antimicrobial drugs requires a complete characterization. The crystal structure of Blp has not been established to date, and its determination was the purpose of this work.

Blp is known to belong to the M23 family of metalloproteases. This family includes zinc–dependent proteases from Gram−positive and Gram−negative bacteria, as well as bacteriophages. The M23 family, in turn, is divided into M23A and M23B subfamilies. The M23A subfamily includes staphylolysin LasA and Blp, as well as AhP protease from *Aeromonas hydrophila* and pseudoalterin from *Pseudoalteromonas* sp. CF6–2 [1,7,8,9,10,11]. The M23B subfamily includes extracellular proteases lysostaphin, Ale−1, SpM23_A, SpM23_B, zoocin A, enterolysin A, autolysins LytM and LytU, and protease gp13 of the bacteriophage ϕ29 [12,13,14,15,16,17,18,19]. A complete list of proteases belonging to the M23 family is available in the MEROPS database https://www.ebi.ac.uk/merops/index.shtml (accessed on 26 October 2022). 

All metalloproteases hydrolyse protein substrates for proteases, and the mechanism of their hydrolysis is known [9,20,21]. Our scientific interest is focused on the ability of metalloproteases of the M23 family, especially Blp, to hydrolyse the peptidoglycan of living target cells. The mechanism of hydrolysis of this complex substrate has yet to be studied. It is possible to get closer to understanding this mechanism only by means of an integrated approach, the obligatory stage of which should be the structural studies of bacteriolytic enzymes.

The structural and functional studies of lytic metalloproteases of this family are actively developing at present [22,23,24,25,26,27]. However, as already mentioned, for bacteriolytic Blp, the structure has not yet been established. A comparison of the architecture of the already established active sites has led to the assumption that M23 metalloproteases are part of a larger metalloenzyme superfamily called LAS (Lysostaphin−type enzymes, D–Ala–D–Ala metallopeptidases, Sonic hedgehog) enzymes, which act mainly as peptidoglycan hydrolases [21,28]. 

Thus, the determination of the Blp structure will add to the characterization of the M23 metalloproteases and contribute to the study of the hydrolysis mechanism of target cells’ peptidoglycan in the future.

## 2. Results

### 2.1. Structural Characterization of Blp from L. capsici VKM B−2533^T^

**Figure 1:**
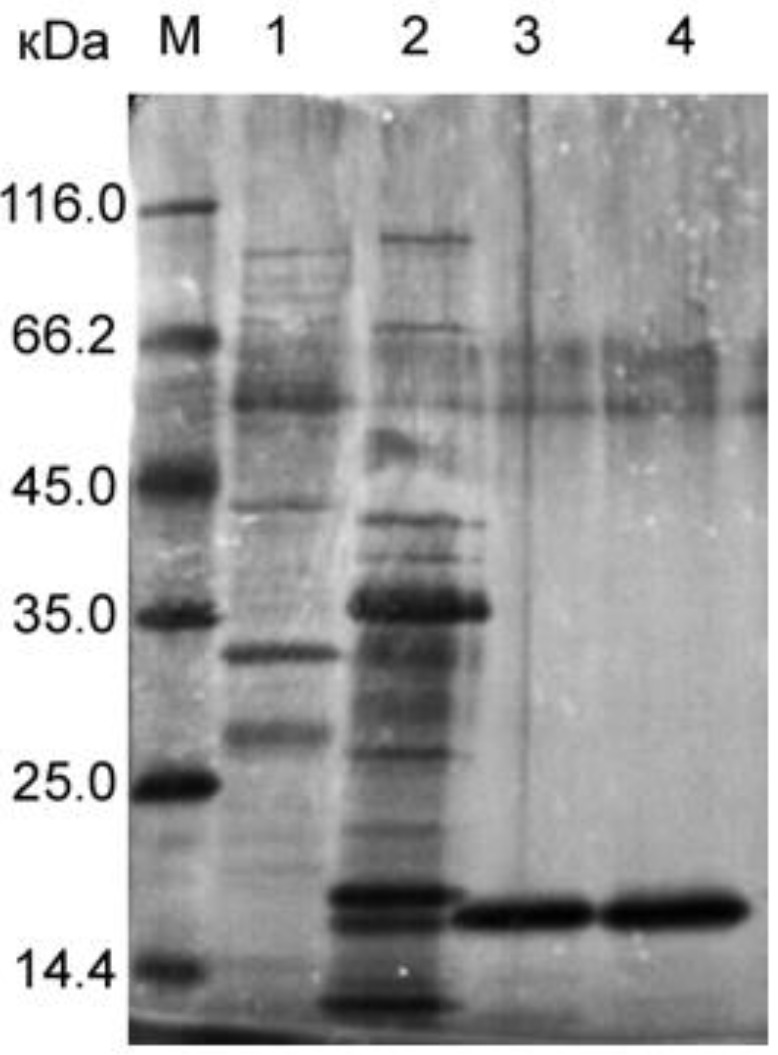
SDS−PAGE: 1, culture fluid of *P. aeruginosa* (12 μL of the preparation); 2, culture fluid of *L. capsici* VKM B−2533^T^ (12 μL of the preparation); 3, LasA (0.084 μg); 4, β−lytic protease (Blp) (0.103 μg).

Blp was isolated from the culture fluid of *L. capsici* VKM B−2533^T^ by the earlier developed purification protocol [5] (Figure 1, lane 4). The concentration of Blp was 0.009 mg/mL. 

**Figure 2:**
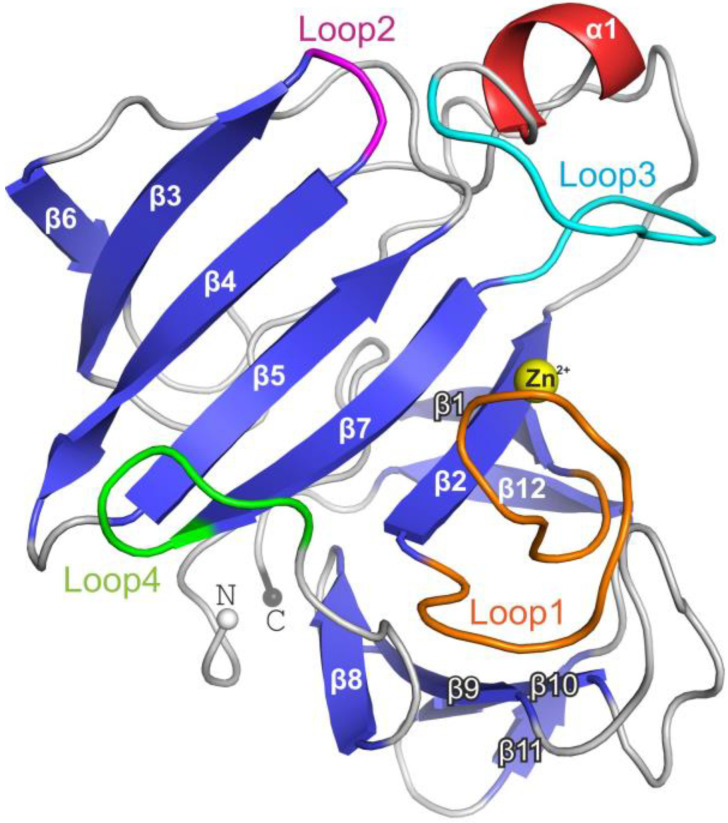
Structure of Blp from *L. capsici* VKM B−2533^T^. The β−sheet core is coloured blue; α−helix is red. Loops 1–4 are shown in different colours.

The Blp structure (Figure 2) was determined at a resolution of 1.57 Å by the molecular replacement technique. The structure of LasA protease from *P. aeruginosa* (PDB ID 3IT5) is also attributed to the M23A family (https://www.ebi.ac.uk/merops/cgi-bin/pepsum?id=M23.002, accessed on 8 November 2022) and was used as a starter model.

Data collection and refinement statistics are presented in Table 1.

**Figure 3:**
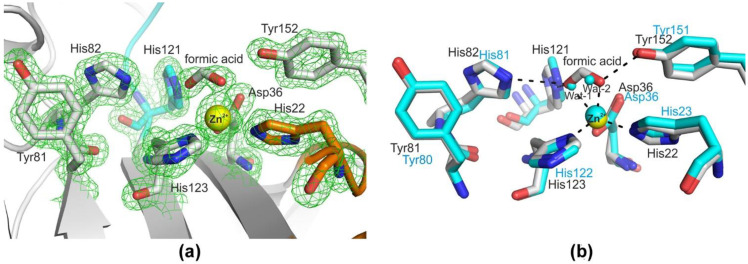
A view of the Blp active site. (**a**) The Blp active site, a fragment of electron density is 2|Fo|−|Fc|, contoured at 1.7σ. Amino acid residues of the loops are coloured as in Figure 2. (**b**) The superimposed active sites of Blp (grey) and LasA (blue). Hydrogen bonding and Zn^2+^–ligand interactions are shown as dashed lines. Zinc ions are rendered as yellow (Blp) and blue (LasA) spheres.

Comparative structural analysis of LasA and Blp showed a high degree of structural homology (rmsd = 1.07 Å) despite the low (44%) amino acid sequence identity of the mature parts of these proteins (Supplementary Figure S2). The overall structure of Blp is shown in Figure 2. The active sites of LasA and Blp were almost the same and represented a groove, the walls of which were formed mainly by four loops (1–4) and the bottom by antiparallel β−sheets (β3–β4–β5–β7–β2). The active site of Blp contained a five−coordinate zinc ion by conserved His 22, His 123, Asp 36, and formic acid, which was replaced by Wat1 and Wat2 in the LasA structure (Figure 3). 

It is generally recognized that the two disulphide bridges and the C−terminal subdomain are unique to the LasA structure [21]. The C−terminal subdomain and disulphide bridge Cys156–Cys169 were preserved in the Blp structure. The conformational lability of the Cys112 side group in the Blp structure was implemented in two positions, one of which did not form the cysteine bridge in 41% of cases. However, this did not lead to any significant changes in the structure of Blp.

The lengths and conformations of the loops in Blp and LasA were very similar: short loops 2 and 4, by two amino acid residues (aa) each; loop 3, by 16 aa; and a difference was observed only in the length of loop 1 (14 aa, in LasA; 15 aa, in Blp).

**Figure 4:**
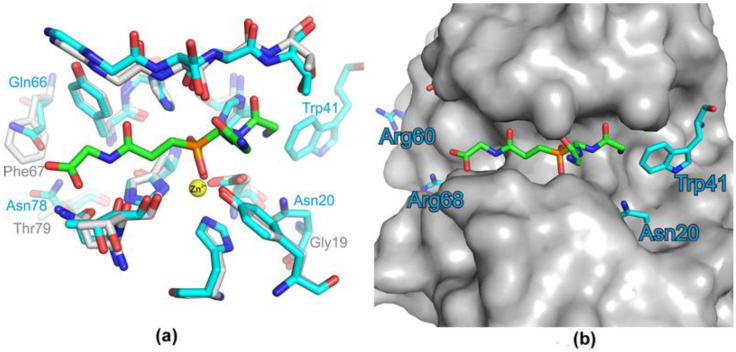
Comparison of the Blp and LasA structures. (**a**) Superimposition of the structures of the LasA (blue) and Blp (grey) active sites with a substrate analogue. Interactions of LasA and Blp with a substrate analogue were modelled based on the structure of LytM catalytic domain with the phosphinic derivative of tetraglycine (PDB ID 4ZYB). The grey sphere indicates the zinc ion. The amino acid residues for LasA and Blp, which may affect the geometry of the active−site groove, are given, respectively, in blue and grey. (**b**) The surface of Blp with superimposed residues of LasA, presented in (**a**), and the substrate analogue, are shown.

In total, seven amino acid residues in the β−strands, and two in loops 1 and 3, differed in the area of the groove that formed the Blp active site; also, there was no amino acid residue for tryptophan with a bulky side group (Table 2, Figure 4). All these substitutions can change the total charge and geometry of the Blp active site compared with that of LasA.

We suggested that the differences found in the Blp active site could lead to differences in the functional properties of this enzyme as compared with LasA.

### 2.2. Comparative Characterization of LasA and Blp Physicochemical and Bacteriolytic Properties

The optimal conditions for the manifestation of bacteriolytic activity against autoclaved *S. aureus* 209P cells by Blp are the low values of ionic strength of a 5.0−mM Britton–Robinson buffer, a pH of 9.0, and a reaction temperature of 55 °C [6]. For LasA, this characterization has not been performed previously. 

**Figure 5:**
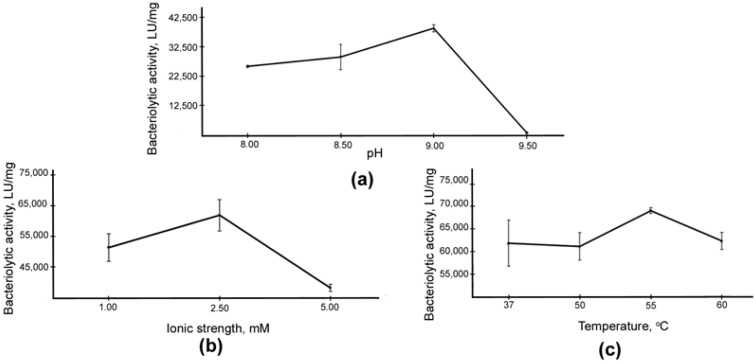
Dependence of the bacteriolytic activity of LasA on some factors: (**a**) pH, (**b**) ionic strength, and (**c**) temperature. The values represent means ± standard deviations of four independent measurements.

The LasA protein was isolated according to our developed protocol from the culture fluid of *P. aeruginosa* (Figure 1, lane 3). The LasA concentration was 0.007 mg/mL. Autoclaved *S. aureus* 209P cells were used as a substrate in studies of the optimal conditions for the manifestation of LasA bacteriolytic activity (Figure 5).

Analysis showed no significant difference in the optimal conditions for the bacteriolytic activity of the enzymes. Thus, for LasA, as for Blp, the optimum values were the low ionic strength of a 2.5−mM Britton–Robinson buffer solution, a pH of 9.0, and a reaction temperature of 55 °C. 

**Figure 6:**
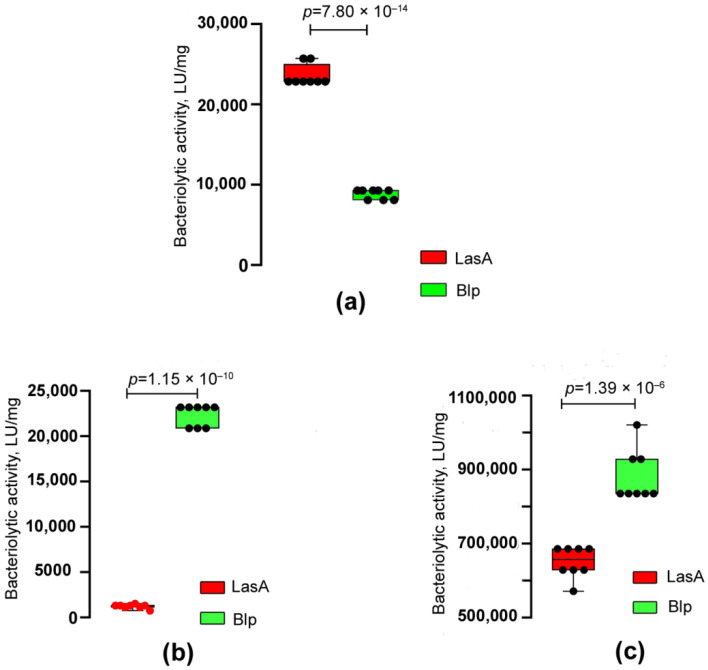
Comparison of the bacteriolytic activities of LasA and Blp with respect to cell substrates. (**a**) Autoclaved cells of *S. aureus* 209P. Statistical analysis was performed using an unpaired two−tailed Student’s *t*−test, *t* = 28.67, *df* = 14. (**b**) Autoclaved cells of *M. luteus* Ac−2230^T^. Statistical analysis was performed using an unpaired two−tailed Student’s *t*−test with Welch’s correction, *t* = 48.82, *df* = 7.5. (**c**) Living cells of *M. luteus* Ac−2230^T^. Statistical analysis was performed using an unpaired two−tailed Student’s *t*−test, *t* = 7.98, *df* = 14. The values were obtained in eight independent experiments. The boxplots show the median and IQR.

The bacteriolytic activities of LasA and Blp with respect to living and autoclaved cells of *S. aureus* 209P, *Kocuria rosea* Ac−2200^T^, and *M. luteus* Ac−2230^T^ were also compared (Table 3, Figure 6). 

As can be seen in Table 3, the bacteriolytic activity of both enzymes against living *S. aureus* 209P cells averages 53,920 LU/mg. At the same time, with respect to autoclaved *S. aureus* 209P cells, the activity of LasA was 2.7 times higher as compared with Blp. With respect to the other target cells, the Blp activity was higher. Thus, it was 1.4 times higher for living *M. luteus* Ac−2230^T^ cells and 18 times higher for autoclaved cells as compared with LasA. Blp had pronounced lytic activity with respect to *K. rosea* Ac−2200^T^, while LasA did not lyse cells of this bacterium at all.

Thus, despite the fact that the physicochemical properties of Blp and LasA do not differ, we revealed differences in their bacteriolytic properties. 

## 3. Discussion

Although Blp was discovered simultaneously with α−lytic protease, it has been less studied until now. However, in practical terms it, should be considered the most promising enzyme for study since it hydrolyses living cells of staphylococci and micrococci with greater efficiency [6]. Structurally, Blp has not been investigated. Earlier, Cruse and Whitaker succeeded in crystallizing Blp, but the structure of the enzyme was not determined [29].

We have recently isolated a Blp from the culture fluid of *L. capsici* VKM B−2533^T^ and characterized it [5]. An efficient homologous expression system for this enzyme was developed [30]. The present work aimed to establish the spatial structure of Blp.

As a result, Blp crystals were obtained, and the structure of this protein was successfully determined at a resolution of 1.57 Å.

The analysis revealed that the Blp structure had a folding characteristic of the M23 family proteases, the LAS superfamily. The enzymes of the LAS superfamily have a similar central folding (core), a four−stranded antiparallel sheet with a conserved active−site topology and architecture, organized around a single divalent metal cation [28]. This is a generalized structure that has a number of features in individual representatives of the LAS superfamily. The active site of these enzymes is formed by four connective loops. One side of the active−site groove is formed by loops 1 and 4, the other by loops 2 and 3. A characteristic representative of this superfamily is LasA *P. aeruginosa* protease, which was almost a complete structural homologue of Blp. The structure of LasA is well known both in a free state (PDB ID 3IT5) and in a complex with tartrate (PDB ID 3IT7). Moreover, the position of the oxygen atoms in the carboxyl group of tartrate corresponed to that of the oxygen atoms of the substrate analogue in the active site [21]. In LasA, the uncomplexed structure of one of the Zn^2+^−bound water molecules of Wat1 and Wat2 (Figure 3b) displaces substrate carbonyl oxygen. A comparative analysis of the structures shows that a ligand, formic acid, was located at the place of these water molecules in the Blp structure (Figure 3). Sodium acetate, which was used in the isolation and crystallization of the protein, contained a formate impurity. The carboxyl group of formate forms the same hydrogen bonds as tartrate in the active site of LasA (PDB ID 3IT7) and the substrate analogue in the structure of the LytM catalytic domain (PDB ID 4ZYB).

Significant differences were observed in the geometry of the active sites’ grooves of these enzymes. Access to the LasA active site was more restricted than the Blp active site due to the bulky charged side groups Arg60, Arg68, Trp41, and Asn20 (Figure 4b). These differences could change the availability of the groove for the substrate and affect the charge of this region. We suggested that the revealed structural differences could also lead to functional differences in these enzymes. 

To confirm the suggestion, we conducted a comparative biochemical characterization of the optimal conditions for the manifestation of the enzymes’ bacteriolytic activity; no significant differences were found. For both enzymes, the optimal values were the low ionic strength of the solution, a pH of 9.0, and a reaction temperature of 55 °C. A comparison of the bacteriolytic activity of the enzymes in relation to living test objects showed Blp to hydrolyse more efficiently not only living *M. luteus* Ac−2230^T^ cells but also living *K. rosea* Ac−2200^T^ cells, which LasA does not hydrolyse at all. With respect to living staphylococcal cells, the activities of the enzymes did not differ. Differences were also found in the efficiency of hydrolysis of autoclaved target cells. Blp was shown to hydrolyse *M. luteus* Ac−2230^T^ cells better and *S. aureus* 209P cells worse. On the whole, the specificity of Blp action against living target cells is broader, and the efficiency of hydrolysis is higher. We would also note here that the type of peptidoglycan of *S. aureus* 209P and *K. rosea* Ac−2200^T^ is A3α (herewith, the interpeptide bridge of *S. aureus* contains 5Gly; and of *K. rosea*, 3−4L−Ala), and of the *M. luteus* Ac−2230^T^ peptidoglycan, A2 (tetrapeptide D−Ala−L−Lys−D−Glu(Gly)−L−Ala in the interpeptide bridge) [31]. It can be assumed that, namely, the differences in the charges of the amino acid residues of the Blp active−site groove as compared with those of LasA determine the interaction of the enzyme with the peptide bridge of *K. rosea* Ac−2200^T^ peptidoglycan, as well as the efficiency of hydrolysis of *M. luteus* Ac−2230^T^. This interesting observation requires further investigation.

A number of papers on the structural and functional aspects of the work of M23 proteases put forward an assumption, based on the modelling of enzyme–substrate interactions, about the influence of the width of the active−site groove and the influence of amino acids forming the walls of this groove on the substrate specificity of LAS enzymes. Thus, in the extracellular protease zoocin A, loops 2 and 4 were longer, and the structure was more open than in lysostaphin, which resulted in a wider binding groove and allowed hydrolysing not only the Gly–Gly bond (which lysostaphin does hydrolyse), but also the D–Ala–L–Ala bond. In the LasA enzyme, loops 1 and 3 were long, and loops 2 and 4 were short. Thus, the groove of the active site was narrowed near Zn^2+^ and wider between loops 2 and 4, which led to a more open structure compared to lysostaphin and broader substrate specificity [21,22,23]. These studies also confirm our observation that the geometry and charge of amino acid residues forming the active−site groove could determine the differences in the manifestation of the bacteriolytic properties of M23 enzymes.

Thus, the Blp structure was determined for the first time. Despite its structural homology with the LasA structure, interesting differences were revealed. Further studies of these differences will enable a better understanding of the mechanism of interaction of Blp and other bacteriolytic enzymes of the M23 family with the peptidoglycan of target cells, which has not yet been established.

## 4. Materials and Methods

### 4.1. Strains and Cultivation Conditions

Strains *L. capsici* VKM B−2533^T^ and *P. aeruginosa* Yaroslavl hospital (a clinical isolate) were cultured on a modified LB medium of the following composition (g/L): peptone, 5; yeast extract, 5; NaCl, 5, pH of 7.5 at 29 °C with a 20−h aeration, and stirring (205 rpm) on a PSU−20i orbital shaker (Biosan, Riga, Latvia).

Strains *S. aureus* 209P, *K. rosea* Ac−2200^T^, and *M. luteus* Ac−2230^T^ were cultured on an agar medium 5/5, developed at the IBPhM RAS, of the following composition (g/L): yeast extract, 1.0; soybean extract, 30.0; tryptone, 5.0; aminopeptide, 60.0; and agar, 15.0, pH 7.2.

### 4.2. Purification of Bacteriolytic Proteins L. capsici VKM B−2533^T^ Blp and P. aeruginosa LasA

The purification of *L. capsici* VKM B−2533^T^ Blp and *P. aeruginosa* LasA was carried out according to our earlier developed protocol [5]. Cells were cultured at 29 °C for 20 h with aeration and then discarded by centrifugation at 5000× *g* and 4 °C for 30 min in an Avanti J−26XP centrifuge (Beckman, Brea, CA, USA). Further precipitation of proteins from the culture fluid was carried out at 80% saturation with ammonium sulphate. Residues of the fractions were produced by centrifugation at 25,960× *g* and 4 °C for 60 min, followed by dialysis against 50 mM Tris−HCl, pH 8.0. The resulting preparation was purified by cation exchange chromatography on a Toyopearl CM−650M column (Tosoh, Tokyo, Japan) equilibrated with 50 mM Tris−HCl, pH 8.0. Elution was carried out with 50 mM Tris−HCl, pH 8.0, containing 0.3 M NaCl. The elution fractions with bacteriolytic activities against living *S. aureus* 209P cells were combined and dialyzed against 50 mM Tris−HCl, pH 8.0. The resulting preparation was applied to an ENrich S column (Bio−Rad, Hercules, CA, USA) equilibrated with the same buffer using an NGC chromatographic system (Bio−Rad, Hercules, CA, USA). Elution was carried out in a linear gradient of 50 mM Tris, pH 8.0, containing 1 M NaCl (from 0 to 1). The final step of purification was carried out by gel filtration on a HiLoad 16/60 (Superdex 75) column (Amersham Biosciences, Uppsala, Sweden) equilibrated with a 30 mM sodium acetate buffer, 0.5 M NaCl, pH 5.5, using an NGC chromatographic system (Bio−Rad, Hercules, CA, USA). The obtained fractions were analysed by SDS−PAGE and by determination of bacteriolytic activity against living *S. aureus* 209P cells, after which the fractions were combined and analysed by MALDI−TOF. The yield of Blp at the purification from the culture fluid of *L. capsici* VKM B−2533^T^ was 2.06 mg/L. The LasA yield at the purification from *P. aeruginosa* culture fluid was 0.06 mg/L.

### 4.3. Protein Measurement by Bradford Method

The total protein concentration in the preparations was measured using the Bradford method [32]. A protocol for the microplates proposed for the proprietary reagent, Coomassie (Thermo Scientific, Waltham, MA, USA), was used to set up the reaction. Absorption was measured at 595 nm on an iMark Microplate Absorption Reader enzyme immunoassay photometer (Bio−Rad, Hercules, CA, USA). The protein concentration was determined by the calibration curve of concentration vs. absorption, constructed for an aqueous solution of BSA (Sigma, Ronkonkoma, NY, USA) within the range of 1–25 µg/mL. 

### 4.4. Determination of Optimal Conditions for LasA Bacteriolytic Activity

The optimal conditions for the bacteriolytic activity of LasA were determined by turbidimetry. As the substrate, we used autoclaved *S. aureus* 209P cells. A 30−mM sodium−acetate buffer, 0.5−M NaCl, pH 5.5 (LasA storage buffer), was used as a control.

To determine the optimal pH value, *S. aureus* 209P cells were dissolved in a 5 mM Britton–Robinson buffer, pH (8.0, 8.5, 9.0, 9.5), to 0.5 OD at 540 nm. The Britton–Robinson buffer contained 0.1 M acetic acid, 0.1 M boric acid, and 0.1 M orthophosphoric acid (adjustment to the required pH value was made by 0.1 M sodium hydroxide) [33]. The LasA preparation was added in a volume of 5–10 µL (0.04–0.07 µg) to a suspension of cells up to 1 mL. The mixture was incubated at 37 °C for 10–30 min.

To determine the optimal value of ionic strength, *S. aureus* 209P cells were dissolved in a 1–5 mM Britton–Robinson buffer, pH 9.0, to 0.5 OD at 540 nm. The LasA preparation was added in a volume of 5 µL (0.04 µg) to a suspension of cells up to 1 mL. The mixture was incubated at 37 °C for 5–10 min.

To determine the optimal value of reaction temperature, *S. aureus* 209P cells were dissolved in a 2.5−mM Britton–Robinson buffer, pH 9.0, to 0.5 OD at 540 nm. The LasA preparation was added in a volume of 5 µL (0.04 µg) to a suspension of cells up to 1 mL. The mixture was incubated at 37–60 °C for 5 min.

The reaction was stopped by placing the test tubes in ice. The absorption of the cell suspension in the samples was measured at 540 nm on a NanoDrop OneC spectrophotometer (Thermo Scientific, Waltham, MA, USA).

A unit of bacteriolytic activity (LU) was taken as an amount of the enzyme, which led to a decrease in absorption of the cell suspension by 0.01 o.u. per 1 min. The value of LU/mL was calculated by the following formula: {[0.5 (initial OD_540_ of suspension) − final OD_540_] × 1000 µL (total reaction volume)}/[5 min (time of reaction) × 5–25 µL (volume of sample) × 0.01 (correction coefficient for OD reduction per min)]. 

### 4.5. Comparison of Bacteriolytic Activity of L. capsici VKM B−2533^T^ Blp and P. aeruginosa LasA Proteins with Respect to Target Cells

To compare the bacteriolytic activities of LasA and Blp with respect to target cells, we also used the turbidimetric method. Living and autoclaved cells of *S. aureus* 209P, *M. luteus* Ac−2230^T^, and *K. rosea* Ac−2200^T^ (living cells only) were suspended in 10 mM Tris−HCl, pH 8.0, to 0.5 OD at 540 nm. The protein preparation was added in a volume of 5–25 µL to a suspension of cells up to 1 mL. The mixture was incubated at 37 °C for 5–30 min. A 30 mM sodium−acetate buffer, 0.5 M NaCl, pH 5.5 (LasA and Blp storage buffer) was used as a control. The reaction was stopped by placing the test tubes in ice. The absorption of the cell suspension in the samples was measured at 540 nm on a NanoDrop OneC spectrophotometer (Thermo Scientific, Waltham, MA, USA).

The bacteriolytic activity (in LU) was calculated as described above. The specific activity of the enzymes was calculated as a ratio of LU per mg of protein. LasA proteins at a concentration of 7.00 µg/mL and Blp proteins at a concentration of 8.62 µg/mL were used in the experiment.

### 4.6. SDS−PAGE

Protein electrophoresis was performed in 12.5% PAG in the presence of SDS by the Laemmli method [34]. 12 µL of drugs were used for the analysis. The samples were heated in a sample buffer at 99 °C for 10 min. A mixture of protein standards (Thermo Fisher Scientific, Waltham, MA, USA) were used as markers: β−galactosidase, 116.0 kDa; BSA, 66.2 kDa; ovalbumin, 45.0 kDa; lactate dehydrogenase, 35.0 kDa; REase Bsp981, 25.0 kDa; β−lactoglobulin, 18.4 kDa; and lysozyme, 14.4 kDa. Electrophoresis in a concentrating gel was performed at 90 V; in a separating gel, at 180 V. Protein bands in the gel were detected using imidazole staining and ZnCl_2_ solutions [35].

### 4.7. MALDI−TOF Mass Spectrometry

MALDI−TOF was performed in accordance with the earlier described method [5].

### 4.8. Crystallization and Crystallography

Blp crystals were obtained by vapour diffusion using a well solution of 400 mM NaCl and 30 mM Na−acetate, pH 5.5. Drops were made by mixing 1 µL Blp (7 mg/mL) in 30 mM Na acetate, pH 5.5, with a 1 µL well solution Crystals grew to maximum dimensions of 15 µm × 15 µm × 250 µm at 297 K. Before freezing in liquid nitrogen for further diffraction data collection, the crystals were transferred into 30% glycerol, 980 mM Na acetate, and 70 mM Na cacodylate, pH 6.5 (Crystal Screen Cryo 7, Hampton Research, Aliso Viejo, CA, USA) as a cryosolution.

Diffraction data were collected on the ID29 beamline at the ESRF electron storage ring (Grenoble, France) using a Pilatus 6M detector (Dectris AG, Baden−Daettwill, Switzerland) [36]. Data were processed and merged using the XDS package [37]. Crystallographic data statistics are summarized in Table 1.

The structures were determined by molecular replacement with Phaser [38] using the structure of a LasA virulence factor from *P. aeruginosa,* determined at 2.0 Å resolution (PDB entry 3IT5), as a search model. Water molecules and metal ions were removed from the model. The initial model was subjected to crystallographic refinement with REFMAC5 [39]. Manual rebuilding of the model was carried out in Coot [40]. The final refinement cycle of the refinement of occupancy with the zinc ion was performed in Phenix [41]. Data and refinement statistics are summarized in Table 1. The atom coordinates and structure factors have been deposited in the Protein Data Bank (PDB ID 8AF1). Figures were prepared using PyMOL [DeLano, W.L. The PyMOL Molecular Graphics System. Available online: http://www.pymol.org/, accessed on 8 November 2022].

### 4.9. Statistical Analysis

Statistical analysis was performed using GraphPad Prism version 8.0.1 (GraphPad Software, San Diego, CA, USA). All experiments were conducted with 4–8 repeats.

The data are presented as means ± standard deviations, as well as in the form of boxplots (medians ± interquartile spans). The data were considered to be significant at *p* < 0.05. 

The normal distribution of the data was verified using the D’Agostino–Pearson complex test. To determine the equality of the variances of two independent groups, the F−test was used for the normally distributed data of two groups, the unpaired two−sided Student’s *t*−test. An unpaired two−tailed Student’s *t*−test with Welch’s correction was used for unequal variances.

## Figures and Tables

**Table 1:** Data collection and refinement statistics.

Data Collection	
Space group	P2_1_2_1_2
a, b, c, Å	70.58, 44.13, 52.70
α = β = γ, °	90.0
Resolution limits, Å	50.0–1.57 (1.61–1.57)
R_sigma_, %	18.5 (111.8)
Mean I/σ (I)	11.16 (2.10)
Completeness, %	100.0 (100.0)
Redundancy	12.53 (12.52)
CC_1/2_, %	99.7 (69.9)
Unique reflections	23 631 (1 701)
**Refinement statistics**	
Resolution, Å	42.23–1.57 (1.64–1.57)
Total number of reflections	23 623 (2 761)
R_work_/R_free_, %	14.8/20.4 (21.8/28.5)
Average B−factor, Å^2^	14.0
**Ramachandran plot**	
Most favourable regions, %	98.9
Allowed regions, %	1.1
**R.m.s. deviations**	
Bond length, Å	0.006
Bond angle, °	0.902

Values in parentheses are for the last resolution shell.

**Table 2:** Amino acid residues, differing in Blp and LasA, in the active−site groove.

Blp	LasA	Location
Gly19	Asn20	Loop1
–	Trp41	
Lys61	Arg60	β3
His63	Leu62	β3
Phe67	Glu66	β4
Glu69	Arg68	β4
Ser77	Ala76	β5
Thr79	Asn78	β5
Asn113	Glu112	Loop3
Lys127	Leu126	β7

**Table 3:** Comparison of the bacteriolytic activities of LasA and Blp with respect to target cells.

Target Cells	Blp, LU/mg	LasA, LU/mg
*S. aureus* 209P living cells *S. aureus* 209P autoclaved cells	52,214 ± 3441 8846 ± 600	55,626 ± 3002 ^ns^ 23,571 ± 1323 ***
*M. luteus* Ac−2230^T^ autoclaved cells	22,332 ± 1201	1238 ± 28 ***
*M. luteus* Ac−2230^T^ living cells *K. rosea* Ac−2200^T^ living cells	888,300 ± 73,020 1817 ± 77	644,898 ± 43,196 *** 0 ***

Results are shown as means ± standard deviations. The mean values were obtained in eight independent experiments. The two groups were compared using an unpaired two−tailed Student’s *t*−test, *** *p* < 0.001 ^ns^, not statistically significant = *p* > 0.05 when comparing the means of the two groups.

## Data Availability

The *L. capsici* VKM B−2533^T^ Blp protein has the locus tag IEQ11_RS04180 (protein id=WP_191821694.1). The gene of the *P. aeruginosa* LasA was entered into the GenBank under accession number OP604556. The atom coordinates and structure factors of the Blp have been deposited in the Protein Data Bank (PDB ID 8AF1). All other relevant data are available from the corresponding author upon request.

## Supplementary Materials

The following supporting information can be downloaded at: https://www.mdpi.com/article/10.3390/ijms232416100/s1.
