# Supplementary material for: Structural and Functional Characterization of β−lytic Protease from Lysobacter capsici VKM B−2533T
Source: Int J Mol Sci. 2022 Dec 17;23(24):16100. doi: 10.3390/ijms232416100 (PMC9783410; doi:10.3390/ijms232416100)
Supplement: Supplementary file 1 [file ijms-23-16100-s001.zip › ijms-2055504-supplementary.pdf]

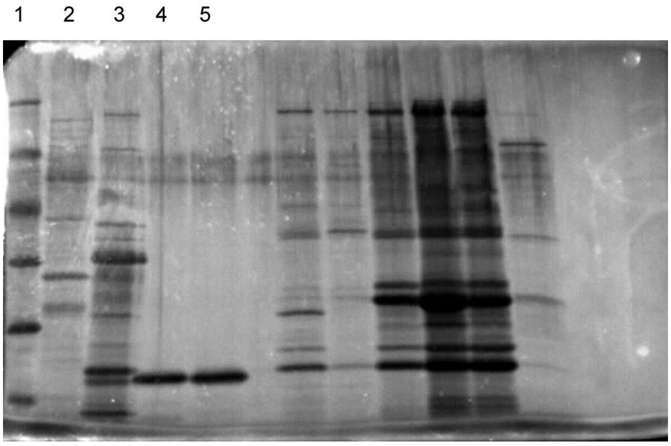

Supplementary Figure S1. Original gel images for Figure 1: lane 1 corresponds to M of Figure 1; lanes 2 and 3, to culture fluid samples of *P. aeruginosa* and *L. capsici* VKM B-2533^T, respectively, of Figure 1; lanes 4 and 5, samples of *P. aeruginosa* LasA and *L. capsici* VKM B-2533^T Blp, respectively, of Figure 1.

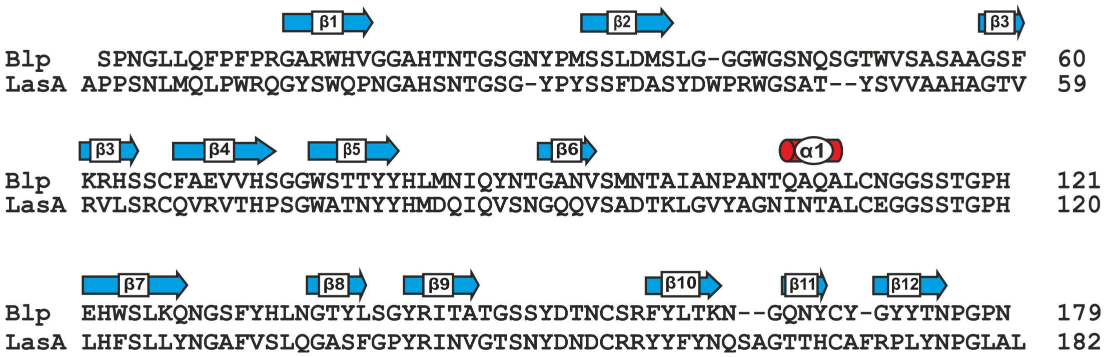

Supplementary Figure S2. Alignment of the spatial structures of *L. capsici* VKM B-2533^T Blp and *P. aeruginosa* LasA.
